# Comparison of treatment plan quality among MRI-based IMRT with a linac, MRI-based IMRT with tri-Co-60 sources, and VMAT for spine SABR

**DOI:** 10.1371/journal.pone.0220039

**Published:** 2019-07-22

**Authors:** Chang Heon Choi, Jin Ho Kim, Jung-in Kim, Jong Min Park

**Affiliations:** 1 Department of Radiation Oncology, Seoul National University Hospital, Seoul, Korea; 2 Institute of Radiation Medicine, Seoul National University Medical Research Center, Seoul, Korea; 3 Biomedical Research Institute, Seoul National University Hospital, Seoul, Korea; 4 Department of Radiation Oncology, Sheikh Khalifa Specialty Hospital, Ras Al Khaimah, United Arab Emirates; 5 Robotics Research Laboratory for Extreme Environments, Advanced Institute of Convergence Technology, Suwon, Korea; University of Nebraska Medical Center, UNITED STATES

## Abstract

**Purpose:**

This study compares the plan quality of magnetic-resonance image (MRI)-based intensity modulated radiation therapy (IMRT) using a linac (MR-linac-IMRT), MRI-based IMRT using tri-Co-60 sources (MR-Co-60-IMRT), and volumetric modulated arc therapy (VMAT) for spine stereotactic ablative radiotherapy (SABR).

**Methods:**

Twenty patients with thoracic spine metastasis were retrospectively selected for this study. For each patient, the MR-linac-IMRT, MR-Co-60-IMRT, and VMAT plans were generated using an identical CT image set and structures, except for the spinal cord and spinal cord planning organ-at-risk volume (PRV). Those two structures were contoured based on CT image sets for VMAT planning while those were contoured based on MR image sets for MR-linac-IMRT and MR-Co-60-IMRT planning. The initial prescription doses were 18 Gy in a single fraction for every plan in this study. If the tolerance level of the spinal cord was not met, the prescription doses were reduced to meet the tolerance level of the spinal cord. Dose-volumetric parameters of each plan were analyzed.

**Results:**

The average spinal cord volumes contoured based on the CT and MR images were 3.8±1.6 cm^3^ and 1.1±1.0 cm^3^, respectively (*p<0*.*001*). For four patients, the prescription doses of VMAT plans were reduced to 16 Gy to satisfy the spinal cord tolerance level. For thirteen patients, the prescription doses of MR-Co-60-IMRT plans were reduced to be less than 16 Gy to meet the spinal cord tolerance level. However, for every MR-linac-IMRT plan, the initial prescription doses of 18 Gy could be delivered to the target volume while satisfying the spinal cord tolerance. The average values of D_10%_, V_10Gy_, and V_14Gy_ of the spinal cord PRV consistently indicated that the doses to the spinal cord PRV in the MR-linac-IMRT plans were the lowest among three types of plans in this study (all with *p*≤0.003).

**Conclusion:**

MR-linac-IMRT appears promising for spine SABR.

## Introduction

Stereotactic ablative radiotherapy (SABR) provides an attractive option for managing spinal metastasis, which occurs within or adjacent to the vertebral bodies and spinal cord [1–3]. For spinal metastasis, SABR can deliver a high biologically equivalent dose (BED) to the target volume by utilizing a high dose per fraction over one to five fractions [4]. This is feasible owing to the ability of SABR to generate steep dose gradients between the target volume and radiosensitive organs, which helps deliver a high prescription dose to the target volume while delivering doses less than their tolerance levels to radiosensitive organs [5]. This highly conformal dose distribution can be accurately delivered to a patient using image guidance. Image guidance also helps reduce the target margin, resulting in the reduction of normal tissue irradiation adjacent to the target volume [6, 7]. Particularly, the importance of image guidance is emphasized for spine SABR, requiring steep dose gradients between the target volume and spinal cord owing to the proximity of the target volume to the spinal cord [6, 8].

Image-guided radiation therapy (IGRT) has been utilized for SABR using various imaging techniques such as megavoltage (MV) or kilovoltage (kV) planar imaging, cone beam computed tomography (CBCT), and computed tomography (CT) [9]. Although conventional IGRT techniques using x-rays are beneficial for the verification and correction of patient setup, these techniques have the potential to deliver considerable imaging doses to patients, which are undesirable [10]. Moreover, the x-ray images exhibit inferior soft tissue contrast compared to magnetic resonance imaging (MRI), which delivers no imaging doses to patients [11]. Specifically, MR imaging is optimal for spine SABR as it can visualize the spinal cord owing to the high signal intensity of the cerebrospinal fluid (CSF), which allows more accurate delineation of the spinal cord than CT imaging [12]. Although CT-MR fusion could allow accurate delineation of the spinal cord, systematic errors in the CT-MR fusion exist, which could be approximately 2 mm [13]. Moreover, it was demonstrated that the margins to account for the intrafractional errors for SABR should be at least 2 mm even though the patient setup was verified with the CBCT and proper immobilization was applied [14]. Therefore, MR image-guided radiation therapy (MR-IGRT) might be an optimal option for spine SABR as the internal anatomy motion of a patient could be monitored and managed with the gated beam delivery with near-real-time cine MR imaging [15–17].

The first commercial MR-IGRT system was the ViewRay system (ViewRay Inc., Oakwood Village, OH) capable of MR imaging with a 0.35-T magnetic field. The treatment beams of the ViewRay system are generated using tri-Co-60 sources to be compatible with the MR imaging system. The clinical performance of the ViewRay system was evaluated in several earlier studies [18–22]. The ViewRay system has of the advantage of MR imaging; however, it has disadvantages such as a low penetrating power as well as large penumbrae of the tri-Co-60 sources and the large leaf width of the multileaf collimator (MLC) system, which is 1.05 cm at the source to axis distance of 105 cm [18]. In a previous study, we reported that the intensity modulated radiation therapy (IMRT) plans with the ViewRay system (MR-Co-60 IMRT) were inferior to the volumetric modulated arc therapy (VMAT) plans with a conventional linac for spine SABR; this was thought to be because of the large penumbrae of the Co-60 sources of the ViewRay system even though the spinal cord volumes of the ViewRay system, which were defined based on MR images, were smaller than those of VMAT [23].

To overcome the disadvantages of the Co-60 sources as well as MLC systems with large leaf widths, a new linac combined with an MR imaging system (MR-linac) has become available with the development of MRIdian Linac (ViewRay Inc., Oakwood Village, OH). The MRIdian Linac can perform step-and-shoot IMRT with 6 MV flattening filter free (FFF) photon beams. In addition, the MLC system in MRIdian Linc is a double-focused and double-stacked system with finer resolution than that of the previous ViewRay system; it can project field sizes of 0.2 cm × 0.4 cm to 27.4 cm × 24.1 cm with the effective MLC width of 0.415 cm on the isoplane located at 90 cm from the source.

The MRIdian Linac system overcame the shortcomings of the previous MR-IGRT system, *i*.*e*., the ViewRay system, and appears capable of maximizing the benefits of MR-IGRT. Therefore, it is unclear whether the plan quality of IMRT with MRIdian Linac (MR-linac IMRT) for spine SABR would be better than that of VMAT. In addition, no study investigated whether the quality of the MR-linac IMRT plans would be actually better than that of the MR-Co-60 IMRT plans. Hence, this study examined the treatment plan quality of MR-linac IMRT, MR-Co-60 IMRT, and VMAT with a TrueBeam STx system equipped with a HD 120^TM^ MLC system (Varian Medical Systems, Palo Alto, CA) for spine SABR. To utilize the advantages of MR-IGRT, the spinal cords of the MR-linac IMRT and MR-Co-60 IMRT plans were delineated using MRI; however, those of the VMAT plans were delineated using CT images. This work benefits any program with those technologies and can help them pick a modality for spine SABR that results in better sparing of the spinal cord.

## Materials and methods

### Patient selection, simulation, and contouring

We retrospectively enrolled 20 patients with thoracic spine metastasis (T9-12) at random after obtaining approval from an institutional review board. Approval had been granted by the institutional review board of Seoul National University Hospital (IRB No.1901-059-1002). This study is a retrospective study using an anonymized patient’s CT image set, which cause minimal risk to the patient. Therefore, this study was granted exemption for informed consent from IRB. Every patient was scanned using the Brilliance CT Big Bore (Philips, Amsterdam, Netherlands) system with a 1.5-mm slice thickness. The target volume of the present study was the clinical target volume (CTV), and the organs at risk (OARs) were defined by a single oncologist. Some OARs from among the spinal cord, kidney, lungs, colon, stomach, and esophagus were selected as OARs in this study according to the tumor location. Each plan did not necessarily have the same set of OARs except the spinal cord since tumor locations varied from T9 to T12 and the OARs varied according to the tumor location. Therefore, only the spinal cord was evaluated as an OAR in this study. In the case of the spinal cord, it was delineated differently according to the treatment technique. As mentioned above, CT images were used to contour the spinal cords for VMAT, however, the spinal cords for MR-linac IMRT and MR-Co-60 IMRT were contoured with MR images acquired with the on-board MR imaging systems in the MRIdian Linac and the ViewRay system, respectively. The planning organ-at-risk volume (PRV) of the spinal cord was generated by adding an isotropic margin of 1.5 mm from the spinal cord.

### VMAT planning for spine SABR

The VMAT plans were created with the Eclipse treatment planning system (Varian Medical Systems, Palo Alto, CA) using 10 MV FFF beams of the TrueBeam STx system with HD 120 MLC system. The VMAT plans were optimized following the RTOG 0631 dose constraints using a photon optimizer algorithm (PO, version 13.7, Varian Medical Systems, Palo Alto, CA) [21]. The dose distributions were calculated using the Acuros XB algorithm (Varian Medical Systems, Palo Alto, CA) with a calculation grid size of 2 mm. During the optimization process, the initial prescription dose to the CTV was set to 18 Gy in a single fraction. If the dose to the spinal cord exceeded the tolerance level of the RTOG 0631 study, the prescription dose was reduced to 16 Gy and optimization was repeated [24]. Every plan was normalized to cover 85% of the CTV volume with 100% of the prescription dose.

### IMRT planning with MR-linac for spine SABR

Identical CT images and structures to those used for VMAT planning were used for MR-linac IMRT planning, except the spinal cord and spinal cord PRV as described above. The initial prescription dose to the CTV was also 18 Gy in a single fraction for MR-linac IMRT planning, identical to VMAT planning. If the dose to the spinal cord exceeded the tolerance level of the RTOG 0631 study, the prescription dose was reduced to 16 Gy and optimization was repeated [21]. Step-and-shoot IMRT plans were generated with 6 MV FFF photon beams of the MR-linac using the treatment planning system of MRIdian Linac, which was the MRIdian system (ViewRay Inc., Oakwood Village, OH). For every patient, nine fields with gantry angles of 0°, 40°, 80°, 120°, 160°, 200°, 240°, 280°, and 320° were used, except for two patients whose plan goals were not accomplished with those nine fields. For those two patients, twelve fields were arranged so that the gantry angles were 0°, 30°, 60°, 90°, 120°, 150°, 180°, 210°, 240°, 270°, 300°, and 330°. The MR-linac IMRT plans were also optimized following the RTOG 0631 dose constraints [24]. The Romeijn optimization algorithm of the MRIdian system was disabled to manually select the number of segments, which was 60. The value of the IMRT efficiency was 0.05. For dose calculation, the number of histories was 2,400,000, and the grid size for dose calculation was 3 mm. The dose distributions were calculated with a 0.35-T magnetic field. Similar to the VMAT plans, every MR-linac IMRT plan was normalized to cover 85% of the CTV volume with 100% of the prescription dose. After dose calculation with the MRIdian system, the calculated dose distributions were exported to the Eclipse system for dose-volumetric analysis of the plans [25].

### IMRT planning with the ViewRay system for spine SABR

Identical CT images and structures to those used for MR-linac IMRT and VMAT planning were also used for MR-Co-60 IMRT planning. The initial prescription dose to the CTV was also 18 Gy in a single fraction for MR-Co-60 IMRT planning. If the dose to the spinal cord exceeded the tolerance level of the RTOG 0631 study, the prescription dose was reduced until the tolerance level of the spinal cord was met and optimization was repeated [21]. Step-and-shoot IMRT plans were generated with the ViewRay system using the MRIdian system. A total of twelve fields from four beam groups were used for MR-Co-60 IMRT plans. The gantry angles of each field were 30°, 150°, and 270° (group 1); 50°, 170°, and 290° (group 2); 70°, 190°, and 310° (group 3); and 100°, 220°, and 340° (group 4). The number of beam segments for each field was set to 60 and the value of IMRT efficiency was set to 0.1. The dose distributions were calculated with a 0.35-T magnetic field and the grid size for dose calculation was 3 mm. Every MR-Co-60 IMRT plan was also normalized to cover 85% of the CTV volume with 100% of the prescription dose. The beam-on time was calculated in the ViewRay system on the assumption that each of three Co-60 sources have a maximum activity of 15,000 Ci Same as MR-linac IMRT planning, after dose calculation with the MRIdian system, the calculated dose distributions were exported to the Eclipse system for dose-volumetric analysis of the plans [25].

### Evaluation of treatment plans

Dose-volumetric parameters calculated from each plan were analyzed to evaluate plan quality. For the CTV, the near-minimum dose (dose received at least 98% volume of the target volume, D_98%_), near-maximum dose (D_2%_), and D_90%_, D_80%_, D_5%_, minimum, maximum, and mean doses were calculated. The conformity index (CI) and homogeneity index (HI) were calculated as follows [26, 27]:

$$Comformity\;index\;(CI)=\frac{Volume\;receiving\;100\%\;of\;the\;prescription\;dose}{Volume\;of\;the\;target\;volume}$$ (1)

$$Homogeneity\;index\;(HI)=\frac{D_{2\%}-D_{98\%}}{mean\;dose}$$ (2)

For the OARs, dose volume histograms (DVHs) were calculated and clinically relevant dose-volumetric parameters were analyzed. For the spinal cord and spinal cord PRV, the volumes receiving at least 14 Gy, (V_14Gy_), V_10 Gy_, and D_10%_ were calculated from each type of plans. For the whole body, the gradient index (GI) proposed by Paddick and Lippitz was calculated as follows [28]:

$$Gradient\;index\;(GI)=\frac{Body\;volume\;receiving\;50\%\;of\;the\;prescription\;dose}{Body\;volume\;receiving\;100\%\;of\;the\;prescription\;dose}$$ (3)

A one-way ANOVA was used to examine the statistical significance of differences in the dose-volumetric parameters among the MR-linac IMRT, MR-Co-60 IMRT, and VMAT plans using the IBM SPSS Statistics 25.0 software (IBM Corporation, Armonk, New York). Differences with *p* values equal to or less than 0.05 were regarded statistically significant in this study.

## Results

### Treatment plan parameters

Average treatment plan parameters of each type of plans are listed in Table 1. The beam-on time of VMAT, MR-linac IMRT, and MR-Co-60 IMRT plans were 4.0 ± 1.1 min, 27.6 ± 5.1 min, and 49.7 ± 11.1 min on average, respectively (*p* < 0.001). The average MU of MR-linac IMRT was approximately three times larger than that of VMAT (16430 ± 3117 MU for MR-linac IMRT and 6037 ± 799 MU for VMAT, *p* < 0.001).

**Table 1:** Average treatment plan parameters.

Parameters	VMAT	MR-linac IMRT	MR-Co-60 IMRT	*p*
Beam-off time (min)	0.02 ± 0.00 (Collimator rotation time)	5.61 ± 0.50 (MLC & gantry movement time)	-	*< 0*.*001*
Beam-on time (min)	4.0 ± 1.1	27.6 ± 5.1	49.7 ± 11.1	*< 0*.*001*
Monitor unit (MU)	6037 ± 799	16430 ± 3117	-	*< 0*.*001*
Number of beam segments	354 (two full arcs)	60 ± 1	60 ± 5	0.264

VMAT: volumetric modulated arc therapy, MR-linac: linear accelerator with magnetic resonance imaging system, IMRT: intensity modulated radiation therapy, MR-Co-60: radiation therapy system using tri-Co-60 sources with magnetic resonance imaging system, MLC: multileaf collimator, MU: monitor unit.

### Dose-volumetric parameters of the target volume

**Fig 1:**
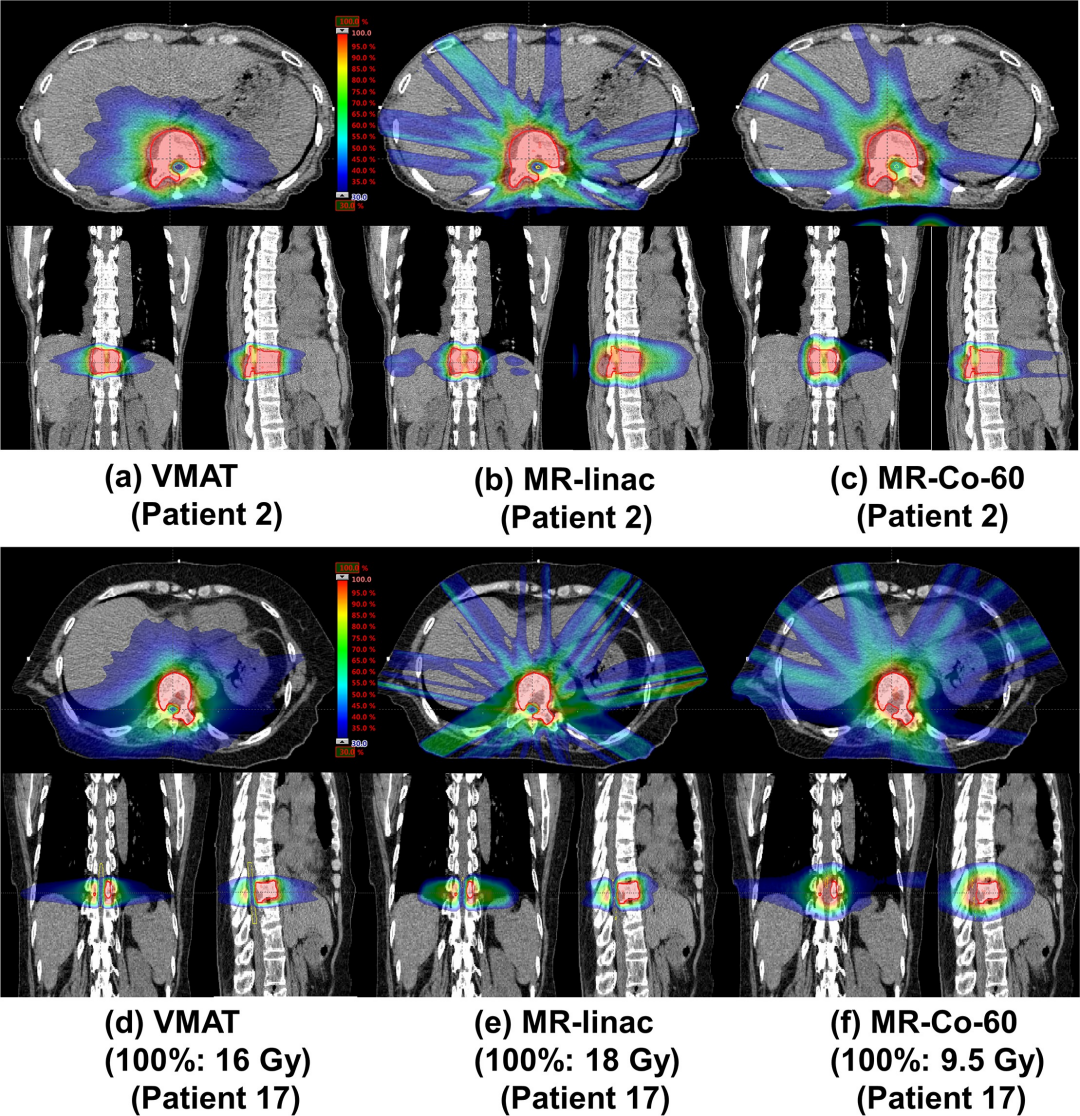
Dose distributions of the volumetric modulated arc therapy (VMAT) plans, intensity modulated radiation therapy (IMRT) plans with the MRIdian Linac system (MR-linac), and IMRT plans with the ViewRay system (MR-Co-60) of two representative cases for spine stereotactic ablative radiotherapy (SABR). One (patient No. 2) is a representative case with a prescription dose of 18 Gy for VMAT (a), MR-linac IMRT (b), and MR-Co-60 IMRT plans (c). The other (patient No. 17) is a representative case with prescription doses of 16 Gy, 18 Gy and 9.5 Gy for VMAT (d), MR-linac IMRT (e), and MR-Co-60 IMRT plans (f), respectively.

**Fig 2:**
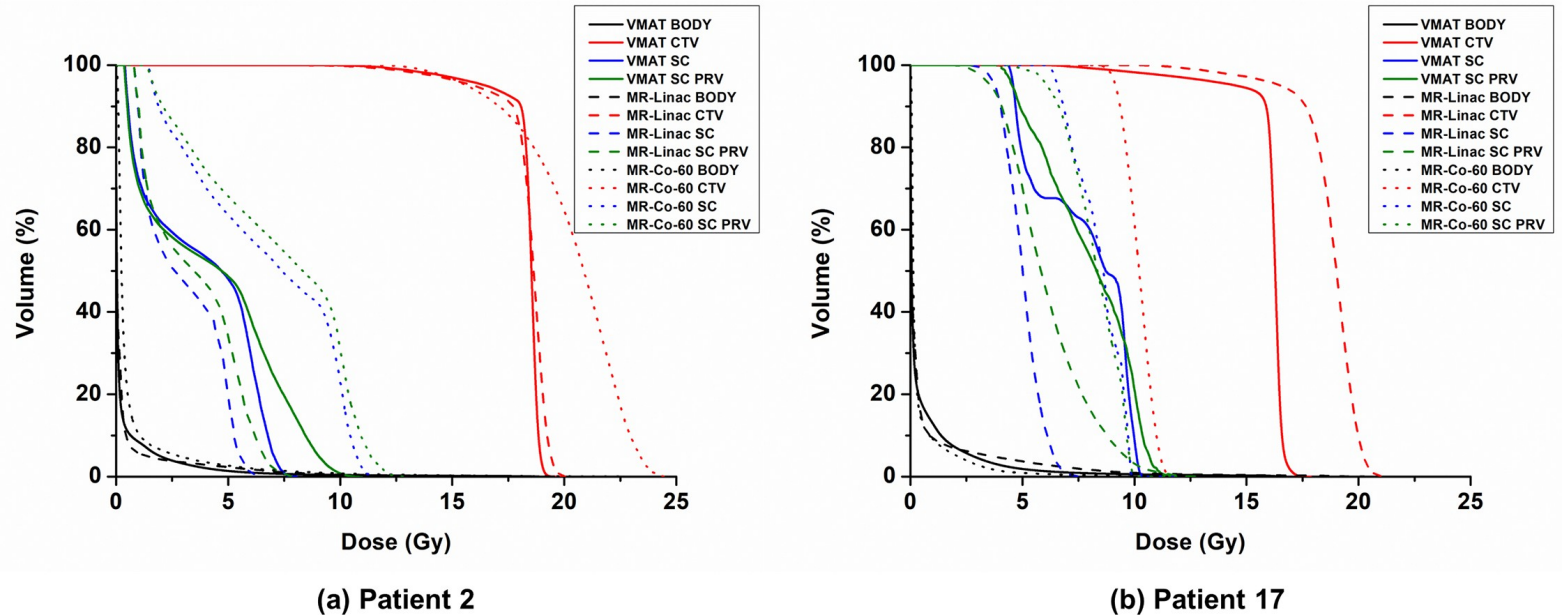
Dose volume histograms (DVHs) of the volumetric modulated arc therapy (VMAT) plans, intensity modulated radiation therapy (IMRT) plans with the MRIdian Linac system (MR-linac), and IMRT plans with the ViewRay system (MR-Co-60) of two representative cases for spine stereotactic ablative radiotherapy (SABR). One (patient No. 2) is a representative case with a prescription dose of 18 Gy for VMAT, MR-linac IMRT, and MR-Co-60 IMRT plans (a). The other (patient No. 17) is a representative case with prescription doses of 16 Gy, 18 Gy and 9.5 Gy for VMAT, MR-linac IMRT, and MR-Co-60 IMRT plans, respectively (b). The DVHs of VMAT, MR-linac IMRT, and MR-Co-60 IMRT plans are plotted as solid, dashed and dotted lines, respectively (black color: whole body, red color: clinical target volume, blue color: spinal cord, and green color: spinal cord planning organ-at-risk volume).

The average dose-volumetric parameters of the CTV are listed in Table 2. For four out of the 20 VMAT patients (patient numbers of 16, 17, 18, and 19), doses to the spinal cord were higher than the tolerance level; therefore, the prescription doses were reduced from 18 Gy to 16 Gy to satisfy the tolerance level of the spinal cord. For the MR-linac IMRT plans, doses to the spinal cord were always less than the tolerance level with the initial prescription dose of 18 Gy even for the four cases mentioned above. Therefore, the prescription doses of MR-linac IMRT plans were always 18 Gy in a single fraction for every patient in this study. For eighteen out of the 20 MR-Co-60 IMRT patients, doses to the spinal cord were higher than the tolerance level; therefore, the prescription doses of those patients were reduced to be less than or equal to 16 Gy (16 Gy prescription doses for five patients and variable prescription doses less than 16 Gy for thirteen patients). Dose distributions of each type of plans of two representative patient cases (patient No. 2 and 17) are illustrated in Fig 1. One is a representative case with a prescription dose of 18 Gy for every type of plans (patient No. 2) and the other is a representative case with a prescription dose of 16 Gy, 18 Gy and 9.5 Gy for the VMAT, MR-linac IMRT, and MR-Co-60 IMRT plans, respectively (patient No. 17). The DVHs of these two patients are plotted in Fig 2.

**Table 2:** Average dose-volumetric parameters of the clinical target volume (CTV) of VMAT, MR-linac IMRT, and MR-Co-60 IMRT plans.

Dose-volumetric parameter	VMAT	MR-linac IMRT	MR Co-60 IMRT	*p*
CTV volume (cm^3^)	57.0 ± 48.8			*-*
D_98%_ (Gy)	15.29 ± 2.30	15.27 ± 1.67	10.89 ± 2.16	*< 0*.*001*
D_90%_ (Gy)	17.45 ± 1.15	17.74 ± 0.65	12.09 ± 3.06	*< 0*.*001*
D_80%_ (Gy)	17.67 ± 0.85	18.16 ± 0.09	12.80 ± 3.66	*< 0*.*001*
D_5%_ (Gy)	18.33 ± 0.84	19.55 ± 0.04	15.06 ± 5.17	*< 0*.*001*
D_2%_ (Gy)	18.48 ± 0.88	19.75 ± 0.31	15.27 ± 5.28	*< 0*.*001*
Minimum dose (Gy)	10.54 ± 0.88	9.95 ± 0.34	9.04 ± 1.46	*< 0*.*001*
Maximum dose (Gy)	19.24 ± 0.89	20.81 ± 0.37	16.11 ± 5.62	*< 0*.*001*
Mean dose (Gy)	17.79 ± 3.18	18.53 ± 2.72	13.63 ± 4.21	*< 0*.*001*
Conformity index (CI)	0.853 ± 0.209	1.027 ± 0.087	1.451 ± 0.551	*< 0*.*001*
Homogeneity index (HI)	0.089 ± 0.046	0.140 ± 0.047	0.282 ± 0.035	*< 0*.*001*

VMAT: volumetric modulated arc therapy, MR-linac: linear accelerator with magnetic resonance imaging system, IMRT: intensity modulated radiation therapy, MR-Co-60: radiation therapy system using tri-Co-60 sources with magnetic resonance imaging system, D_n%_: dose received by *n*% volume of the CTV.

The average values of the maximum dose, mean dose, D_90%_, D_80%_, D_5%_, and D_2%_ consistently indicated that the doses to the CTV of the MR-linac IMRT plans were the highest among every type of plans (all with *p* < 0.001). The target conformity of the MR-linac IMRT plans was the best among every type of plans on average (CI = 0.853 ± 0.209, 1.027 ± 0.087 and 1.451 ± 0.551 for VMAT, MR-linac IMRT, and MR-Co-60 IMRT plans, respectively, *p <* 0.001); however, the dose uniformity inside the target volume of VMAT plans was the best among every type of plans on average (HI = 0.089 ± 0.046, 0.140 ± 0.047, 0.282 ± 0.035 for VMAT, MR-linac IMRT, and MR-Co-60 IMRT, respectively, *p* < 0.001).

### Dose-volumetric parameters of the spinal cord and whole body

Table 3 presents the average dose-volumetric parameters of the spinal cord, spinal cord PRV, and whole body of each type of plans. The average volume of the spinal cord delineated with CT images for VMAT planning was 3.8 ± 1.6 cm^3^ while that delineated with MR images was 1.1 ± 1.0 cm^3^ (*p <* 0.001).

**Table 3:** Average dose-volumetric parameters of the spinal cord, spinal cord PRV, and whole body of VMAT, MR-linac IMRT, and MR-Co-60 IMRT plans.

Dose-volumetric parameter	VMAT	MR-linac IMRT	MR Co-60 IMRT	*p*
Spinal cord				
Volume (cm^3^)	3.8 ± 1.6	1.1 ± 1.0		*< 0*.*001*
D_10%_ (Gy)	8.57 ± 1.69	7.99 ± 2.45	9.80 ± 1.56	0.020
V_10Gy_ (cm^3^)	0.2 ± 0.1	0.1 ± 0.2	0.1± 0.2	0.279
V_14Gy_ (cm^3^)	0.0 ± 0.0	0.0 ± 0.0	0.0 ± 0.0	0.145
Maximum dose (Gy)	11.39 ± 2.15	9.38 ± 3.13	10.40 ± 1.59	*0*.*024*
Spinal cord PRV				
Volume (cm^3^)	8.0 ± 2.7	3.2 ± 1.9		*< 0*.*001*
D_10%_ (Gy)	10.99 ± 2.69	9.81 ± 1.69	10.07 ± 1.45	*< 0*.*001*
V_10Gy_ (cm^3^)	1.4 ± 1.2	0.2 ± 0.5	0.5 ± 0.6	*< 0*.*001*
V_14Gy_ (cm^3^)	0.2 ± 0.2	0.0 ± 0.1	0.0 ± 0.0	*0*.*003*
Maximum dose (Gy)	15.02 ± 2.36	12.24 ± 4.37	10.98 ± 1.58	*< 0*.*001*
Whole body				
V_50%_ (cm^3^)	240.1 ± 172.2	325.5 ± 256.2	501.2 ± 485.7	0.049
V_100%_ (cm^3^)	56.4 ± 45.2	60.5 ± 54.9	76.8 ±73.4	*< 0*.*001*
Gradient index (GI)	4.49 ± 0.64	5.74 ± 1.01	6.85 ±1.56	*< 0*.*001*

PRV: planning organ-at-risk volume, VMAT: volumetric modulated arc therapy, MR-linac: linear accelerator with magnetic resonance imaging system, IMRT: intensity modulated radiation therapy, MR-Co-60: radiation therapy system using tri-Co-60 sources with magnetic resonance imaging system, D_n%_: dose received by *n*% volume of a structure, V_nGy_ (V_n%_): volume receiving more than *n* Gy (or *n*% of the prescription dose) of a structure.

The average values of the maximum doses and D_10%_ of the spinal cord in the MR-linac IMRT plans were the lowest among every type of plans (the average values of the maximum doses were 11.39 ± 2.15 Gy for VMAT, 9.38 ± 3.13 Gy for MR-linac IMRT, and 10.40 ± 1.59 Gy for MR-Co-60 IMRT with *p* = 0.024 and the average values of the D_10%_ were 8.57 ± 1.69 Gy for VMAT, 7.99 ± 2.45 Gy for MR-linac IMRT, and 9.80 ± 1.56 Gy for MR-Co-60 IMRT with *p* = 0.020).

For the spinal cord PRV, the average values of D_10%_ V_10Gy_, V_14Gy_, and the maximum dose consistently indicated that the doses to the spinal cord PRV in the MR-linac IMRT were the lowest than the others (all with *p* ≤ 0.003).

Particularly, for patient No. 17, even though the prescription dose in the MR-linac IMRT plan was higher than those in the VMAT and MR-Co-60 IMRT plans (18 Gy for MR-linac IMRT plan, 16 Gy for VMAT plan, and 9.5 Gy for MR-Co-60 IMRT plan), the values of V_10Gy_ and V_14Gy_ of both the spinal cord and the spinal cord PRV in the MR-linac IMRT plan were the lowest among every type of plans.

For the whole body, the average values of V_50%_, V_100%_, and GI consistently indicated that the normal tissue irradiation of the VMAT plans was less than the others (all with *p* < 0.05).

## Discussion

In the present study, the treatment plan quality of the MR-linac IMRT was compared to those of the VMAT and MR-Co-60 IMRT plans. Since the VMAT technique with a FFF photon beam is known to be the best option for spine SABR with a conventional linac owing to its superior target conformity and treatment efficiency [2, 23, 27], therefore, in this study, we compared MR-IGRT IMRT plans (*i*.*e*., MR-linac IMRT and MR-Co-60 IMRT plans) to the VMAT plans with a conventional linac not the IMRT plans with a conventional linac to identify which radiotherapy technique would be the best option for spine SABR currently. To review the results, MR-linac IMRT plans showed the better performance than VMAT and MR-Co-60 IMRT plans in terms of sparing of doses to the spinal cord while delivering prescription doses equal to or even larger than those in the VMAT and MR-Co-60 IMRT plans. To compare the MR-linac IMRT plans with the MR-Co-60 IMRT plans, the better performance of the MR-linac IMRT than that of the MR-Co-60 IMRT is reasonable since the treatment beam delivery system of the MRIdian Linac is superior to that of the ViewRay system (effective MLC width of 4.15 mm for the MRIdian Linac vs. MLC width of 10.5 mm for the ViewRay system and 4.8 mm penumbra at 10 cm depth for the MRIdian Linac vs. 18.8 mm penumbra at 10 cm depth for the ViewRay system) [23, 29, 30]. Although both the MR-linac IMRT and the MR-Co-60 IMRT plans were generated with identical small spinal cords delineated based on the MR images, superior beam delivery system of the MRIdian Linac to that of the ViewRay system enabled better plan quality of the MR-linac IMRT than that of the MR-Co-60 IMRT. To compare the MR-linac IMRT plans with the VMAT plans, the MR-linac IMRT showed better performance than that of VMAT for spine SABR considering doses delivered to the spinal cord as well as spinal cord PRV owing to the spinal cord volume reduction of the MR-linac IMRT by MR-guidance. However, considering the whole body, irradiation of normal tissue by intermediate doses (50% of the prescription dose) of VMAT plans was smaller than that of MR-linac IMRT plans (the smallest values of V_50%_ and GI of the whole body). As shown in Fig 1, the MR-linac IMRT plans (as well as the MR-Co-60 IMRT plans) showed intermediate-dose streaks in the body more frequently than did the VMAT plans. Since VMAT delivers beams through arcs around a patient, *i*.*e*., VMAT delivers beams from a wide variety of directions around a patient, in general, intermediate-doses are not concentrated at particular regions in the body for VMAT. On the contrary, IMRT delivers beams from fewer directions around a patient compared to VMAT, doses from each field could be piled up at particular regions in the body and the dose streaks could be generated.

In terms of treatment efficiency, although the MR-linac IMRT plans were better than the MR-Co-60 IMRT plans, the MR-linac IMRT plans were poorer than the VMAT plans, showing much higher MUs and treatment times [31]. This is natural as the MR-linac IMRT is step-and-shoot IMRT requiring a much higher treatment time than that for VMAT. Moreover, the maximum dose rate of the TrueBeam STx system is 2400 cGy/min for a 10 MV FFF photon beam at depth of dose maximum while that of the MRIdian Linac system is 600 cGy/min at maximum dose depth (a four times higher dose rate of VMAT than that of MR-linac IMRT). The long treatment times of the MR-linac IMRT and the MR-Co-60 IMRT would cause discomfort to patients during treatment. Apart from the discomfort caused to patients, there might be an increase in the intrafractional errors owing to the long treatment time [32]. However, this would not be the case in MR-linac IMRT as well as MR-Co-60 IMRT since the intrafractional motion of a patient would be monitored and managed by the gated beam delivery based on the near-real-time cine MR images although the treatment time would increase.

Various planning studies with the ViewRay system previously performed reported more favorable or at least comparable plan qualities with the ViewRay system compared to those with the conventional linac-based IMRT or linac-based VMAT for various tumor sites [18, 19, 33, 34]; however, only for spine SABR, it was reported that the IMRT plan quality with ViewRay system was much poorer than that of VMAT [23]. Similarly, in the present study, the MR-Co-60 IMRT plans also showed the worse plan quality than the others for spine SABR although the MR-Co-60 IMRT plans were generated with the small spinal cords delineated based on the MR images [23, 29]. It seems not advisable to perform spine SABR with the MR-Co-60 IMRT technique.

Although MR-linac IMRT plans showed intermediate-dose streaks in the body, only the MR-linac IMRT technique could deliver the initial prescription dose of 18 Gy to every enrolled patient in this study. In addition, MR-linac IMRT is more beneficial for spine SABR than VMAT as the intrafractional internal anatomy motions can be monitored and managed during treatment with the gated beam delivery based on the near-real-time cine MR images; this can guarantee accurate deliveries of the intended dose distributions to patients [29]. According to the results of the present study, the MRIdian Linac system appears to maximize the benefits of MR-IGRT by overcoming the disadvantages of the previous version of the MR-IGRT system, the ViewRay system. Therefore, MR-linac IMRT appears promising for spine SABR although its clinical efficacy should be verified with a clinical trial.

## Supporting information

S1 Table Dose-volumetric parameters of volumetric modulated arc therapy (VMAT) plans, magnetic-resonance (MR) image-guided intensity modulated radiation therapy (IMRT) using a linac (MR-linac IMRT) plans, and MR image-guided IMRT using tri-Co-60 sources (MR-Co-60 IMRT) plans are shown for each patient. (XLSX)

## References

[1] Sahgal A, Larson DA, Chang EL. Stereotactic body radiosurgery for spinal metastases: a critical review. Int J Radiat Oncol Biol Phys. 2008;71(3):652–65. 10.1016/j.ijrobp.2008.02.060 18514775

[2] Gallo JJ, Kaufman I, Powell R, Pandya S, Somnay A, Bossenberger T, et al Single‐fraction spine SBRT end‐to‐end testing on TomoTherapy, Vero, TrueBeam, and CyberKnife treatment platforms using a novel anthropomorphic phantom. J Appl Clin Med Phys. 2015;16(1):170–82. 10.1120/jacmp.v16i1.5120 PMC5689980 25679169

[3] Thibault I, Campbell M, Tseng C-L, Atenafu EG, Letourneau D, Yu E, et al Salvage stereotactic body radiotherapy (SBRT) following in-field failure of initial SBRT for spinal metastases. Int J Radiat Oncol Biol Phys. 2015;93(2):353–60. 10.1016/j.ijrobp.2015.03.029 26383680

[4] Redmond KJ, Lo SS, Fisher C, Sahgal A. Postoperative stereotactic body radiation therapy (SBRT) for spine metastases: a critical review to guide practice. Int J Radiat Oncol Biol Phys. 2016;95(5):1414–28. 10.1016/j.ijrobp.2016.03.027 27479724

[5] Katsoulakis E, Kumar K, Laufer I, Yamada Y. Stereotactic body radiotherapy in the treatment of spinal metastases. Semin Radiat Oncol. 2017;27(3):209–17. 10.1016/j.semradonc.2017.03.004 28577828

[6] Yamada Y, Bilsky MH, Lovelock DM, Venkatraman ES, Toner S, Johnson J, et al High-dose, single-fraction image-guided intensity-modulated radiotherapy for metastatic spinal lesions. Int J Radiat Oncol Biol Phys. 2008;71(2):484–90. 10.1016/j.ijrobp.2007.11.046 18234445

[7] Keall PJ, Nguyen DT, O'Brien R, Zhang P, Happersett L, Bertholet J, et al Review of real-time 3-dimensional image guided radiation therapy on standard-equipped cancer radiation therapy systems: Are we at the tipping point for the era of real-time radiation therapy? Int J Radiat Oncol Biol Phys. 2018;102(4):922–31. 10.1016/j.ijrobp.2018.04.016 29784460 PMC6800174

[8] Chang Z, Wang Z, Ma J, O’Daniel JC, Kirkpatrick J, Yin F-F. 6D image guidance for spinal non-invasive stereotactic body radiation therapy: Comparison between ExacTrac X-ray 6D with kilo-voltage cone-beam CT. Radiother Oncol. 2010;95(1):116–21. 10.1016/j.radonc.2009.12.036 20122747

[9] Boda-Heggemann J, Lohr F, Wenz F, Flentje M, Guckenberger M. kV cone-beam CT-based IGRT. Strahlenther Onkol. 2011;187(5):284–91. 10.1007/s00066-011-2236-4 21533757

[10] Murphy MJ, Balter J, Balter S, BenComo JA, Das IJ, Jiang SB, et al The management of imaging dose during image‐guided radiotherapy: report of the AAPM Task Group 75. Med Phys. 2007;34(10):4041–63. 10.1118/1.2775667 17985650

[11] Steiner E, Stock M, Kostresevic B, Ableitinger A, Jelen U, Prokesch H, et al Imaging dose assessment for IGRT in particle beam therapy. Radiother Oncol. 2013;109(3):409–13. 10.1016/j.radonc.2013.09.007 24128802

[12] Dahele M, Zindler JD, Sanchez E, Verbakel WF, Kuijer JP, Slotman BJ, et al Imaging for stereotactic spine radiotherapy: clinical considerations. Int J Radiat Oncol Biol Phys. 2011;81(2):321–30. 10.1016/j.ijrobp.2011.04.039 21664062

[13] Winey B, Al-Halabi H, DeLaney T, Guckenberger M, Mantel F, Sheehan J, et al Variability of Spinal Cord Definition Using MR T2 Versus CT Myelography for Spine Radiosurgery. Int J Radiat Oncol Biol Phys. 2015;93(3):S159–S60.

[14] Li W, Sahgal A, Foote M, Millar B-A, Jaffray DA, Letourneau D. Impact of immobilization on intrafraction motion for spine stereotactic body radiotherapy using cone beam computed tomography. Int J Radiat Oncol Biol Phys. 2012;84(2):520–6. 10.1016/j.ijrobp.2011.12.039 22401920

[15] Pollard JM, Wen Z, Sadagopan R, Wang J, Ibbott GS. The future of image-guided radiotherapy will be MR guided. Br J Radiol. 2017;90(1073):20160667 10.1259/bjr.20160667 28256898 PMC5605101

[16] Bjerre T, Crijns S, af Rosenschöld PM, Aznar M, Specht L, Larsen R, et al Three-dimensional MRI-linac intra-fraction guidance using multiple orthogonal cine-MRI planes. Phys Med Biol. 2013;58(14):4943 10.1088/0031-9155/58/14/4943 23807514

[17] Tseng C-L, Sussman MS, Atenafu EG, Letourneau D, Ma L, Soliman H, et al Magnetic resonance imaging assessment of spinal cord and cauda equina motion in supine patients with spinal metastases planned for spine stereotactic body radiation therapy. Int J Radiat Oncol Biol Phys. 2015;91(5):995–1002. 10.1016/j.ijrobp.2014.12.037 25832691

[18] Park JM, Park S-Y, Kim HJ, Wu H-G, Carlson J, Kim J-i. A comparative planning study for lung SABR between tri-Co-60 magnetic resonance image guided radiation therapy system and volumetric modulated arc therapy. Radiother Oncol. 2016;120(2):279–85. 10.1016/j.radonc.2016.06.013 27401404

[19] Ramey SJ, Padgett KR, Lamichhane N, Neboori HJ, Kwon D, Mellon EA, et al Dosimetric analysis of stereotactic body radiation therapy for pancreatic cancer using MR-guided Tri-60Co unit, MR-guided LINAC, and conventional LINAC-based plans. Pract Radiat Oncol. 2018;8(5):e312–e21. 10.1016/j.prro.2018.02.010 29703704

[20] Wooten HO, Green O, Yang M, DeWees T, Kashani R, Olsen J, et al Quality of intensity modulated radiation therapy treatment plans using a ^60^Co magnetic resonance image guidance radiation therapy system. Int J Radiat Oncol Biol Phys. 2015;92(4):771–8. 10.1016/j.ijrobp.2015.02.057 26104932

[21] Kishan AU, Cao M, Wang P-C, Mikaeilian AG, Tenn S, Rwigema J-CM, et al Feasibility of magnetic resonance imaging–guided liver stereotactic body radiation therapy: A comparison between modulated tri-cobalt-60 teletherapy and linear accelerator–based intensity modulated radiation therapy. Pract Radiat Oncol. 2015;5(5):330–7. 10.1016/j.prro.2015.02.014 25823383

[22] Son J, An HJ, Choi CH, Chie EK, Kim JH, Park JM, et al Assessment of Dose Distributions According to Low Magnetic Field Effect for Prostate SABR. J Radiat Prot and Res. 2019;44(1):26–31.

[23] Choi CH, Park S-Y, Kim J-i, Kim JH, Kim K, Carlson J, et al Quality of tri-Co-60 MR-IGRT treatment plans in comparison with VMAT treatment plans for spine SABR. Br J Radiol. 2017;90(1070):20160652 10.1259/bjr.20160652 27781486 PMC5685120

[24] Ryu S, Pugh SL, Gerszten PC, Yin F-F, Timmerman RD, Hitchcock YJ, et al RTOG 0631 phase 2/3 study of image guided stereotactic radiosurgery for localized (1–3) spine metastases: phase 2 results. Pract Radiat Oncol. 2014;4(2):76–81. 10.1016/j.prro.2013.05.001 24890347 PMC3711083

[25] Kim J-i, Han JH, Choi CH, An HJ, Wu H-G, Park JM. Discrepancies in Dose-volume Histograms Generated from Different Treatment Planning Systems. J Radiat Prot and Res. 2018;43(2):59–65.

[26] Shaw E, Kline R, Gillin M, Souhami L, Hirschfeld A, Dinapoli R, et al Radiation Therapy Oncology Group: radiosurgery quality assurance guidelines. Int J Radiat Oncol Biol Phys. 1993;27(5):1231–9. 10.1016/0360-3016(93)90548-a 8262852

[27] Zhang P, Happersett L, Hunt M, Jackson A, Zelefsky M, Mageras G. Volumetric modulated arc therapy: planning and evaluation for prostate cancer cases. Int J Radiat Oncol Biol Phys. 2010;76(5):1456–62. 10.1016/j.ijrobp.2009.03.033 19540062

[28] Paddick I, Lippitz B. A simple dose gradient measurement tool to complement the conformity index. J Neurosurg. 2006;105(Supplement):194–201. 10.3171/sup.2006.105.7.194 18503356

[29] Low DA. MRI guided radiotherapy Advances in radiation Oncology: Springer; 2017 p. 41–67.

[30] ViewRay, Inc. Treatment delivery performance ViewRay website. 2018 [cited 13 May 2019]. In: ViewRay website [internet]. Oakwood Village, ViewRay 2018. Available from: http://viewray.businesscatalyst.com/treatment-delivery-performance

[31] Wu QJ, Yoo S, Kirkpatrick JP, Thongphiew D, Yin F-F. Volumetric arc intensity–modulated therapy for spine body radiotherapy: comparison with static intensity-modulated treatment. Int J Radiat Oncol Biol Phys. 2009;75(5):1596–604. 10.1016/j.ijrobp.2009.05.005 19733447

[32] Matsuo Y, Verellen D, Poels K, Mukumoto N, Depuydt T, Akimoto M, et al A multi-centre analysis of treatment procedures and error components in dynamic tumour tracking radiotherapy. Radiother Oncol. 2015;115(3):412–8. 10.1016/j.radonc.2015.05.003 25998806

[33] Park JM, Park SY, Choi CH, Chun M, Kim JH, Kim JI. Treatment plan comparison between Tri-Co-60 magnetic-resonance image-guided radiation therapy and volumetric modulated arc therapy for prostate cancer. Oncotarget. 2017;8(53):91174–84. 10.18632/oncotarget.20039 29207634 PMC5710914

[34] Park JM, Park SY, Kim JI, Kang HC, Choi CH. A comparison of treatment plan quality between Tri-Co-60 intensity modulated radiation therapy and volumetric modulated arc therapy for cervical cancer. Phys Med. 2017;40:11–6. 10.1016/j.ejmp.2017.06.018 28760506

